# Load-displacement experimental data from axial tensile loading of CFRP-SPCC hybrid laminates

**DOI:** 10.1016/j.dib.2020.105306

**Published:** 2020-02-19

**Authors:** Muhammad Akhsin Muflikhun, Alvin Y. Chua

**Affiliations:** a Department of Mechanical and Industrial Engineering, Gadjah Mada University, Indonesia; b Mechanical Engineering Department, De La Salle University, Philippines

**Keywords:** Tensile loading, Hybrid laminate, CFRP, SPCC, Load-displacement

## Abstract

The current paper shows a data set of load-displacement output from axial tensile loading of CFRP-SPCC hybrid laminates. The specimen geometries are cut based on standard procedure from ASTM D-3039. At least 3 positions in each specimen, we measured its width and thickness. Data of the load and displacement were repeated at least 3 samples in each combination of hybrid laminates. Tensile test was conducted with a 1 mm/min of loading rate. The data were recorded from unloading until failure of specimens. The data gives information about the highest load and the behavior of load-displacement in axial tensile loading. By using width and thickness, normalized data can be obtained, the load can be calculated into stress (MPa) unit. The data are useful for researchers and structural engineers that deals with CFRP, SPCC, and hybrid CFRP-SPCC laminates.

**Table:** Specifications Table

Subject	Engineering
Specific subject area	Hybrid material, Mechanics of composite materials, Axial tensile loading, Material properties
Type of data	1. Tables 2. Figures
How data were acquired	Data were acquired from Universal Testing Machine (UTM) and the output are load-displacement value.
Data format	Raw and analyzed
Parameters for data collection	Raw data from UTM output are Load in Newton (N) and displacement in millimeters (mm). Additional parameter added from sample measurement such as thickness, width, and length.
Description of data collection	1. Data were formed as load - displacement tables. 2. Specimen measurement (width and thickness) was measured before the specimen being tested. 3. Material properties that obtained from tensile loading can be transformed to stress and the information can give information about material strength.
Data source location	Data were obtained from the Aoki-Yokozeki lab, department of Aeronautics and Astronautics, The University of Tokyo, Japan.
Data accessibility	With the article
Related research article	The data are related to two previous research papers: 1. https://doi.org/10.1016/j.compstruct.2019.03.094 2. https://doi.org/10.1016/j.compositesb.2019.05.049

**Table:** 

**Value of the Data** • The data presented in the current study provide a complete material performance during axial tensile loading of CFRP laminates and hybrid laminates. • The data can be used by designers, engineers, and scientists to predict the material strength, and maximum load of CFRP, SPCC, and CFRP-SPCC hybrid laminates. • The data of CFRP-SPCC hybrid laminate can be used in several fields such as structural applications (buildings, bridges, towers) and automotive industries. • Since load-displacement is raw data, data processing can be done with different purposes to obtain material strength, stiffness, and ABD matrixes of hybrid laminates.

## 1 Data description

**Fig. 1:**
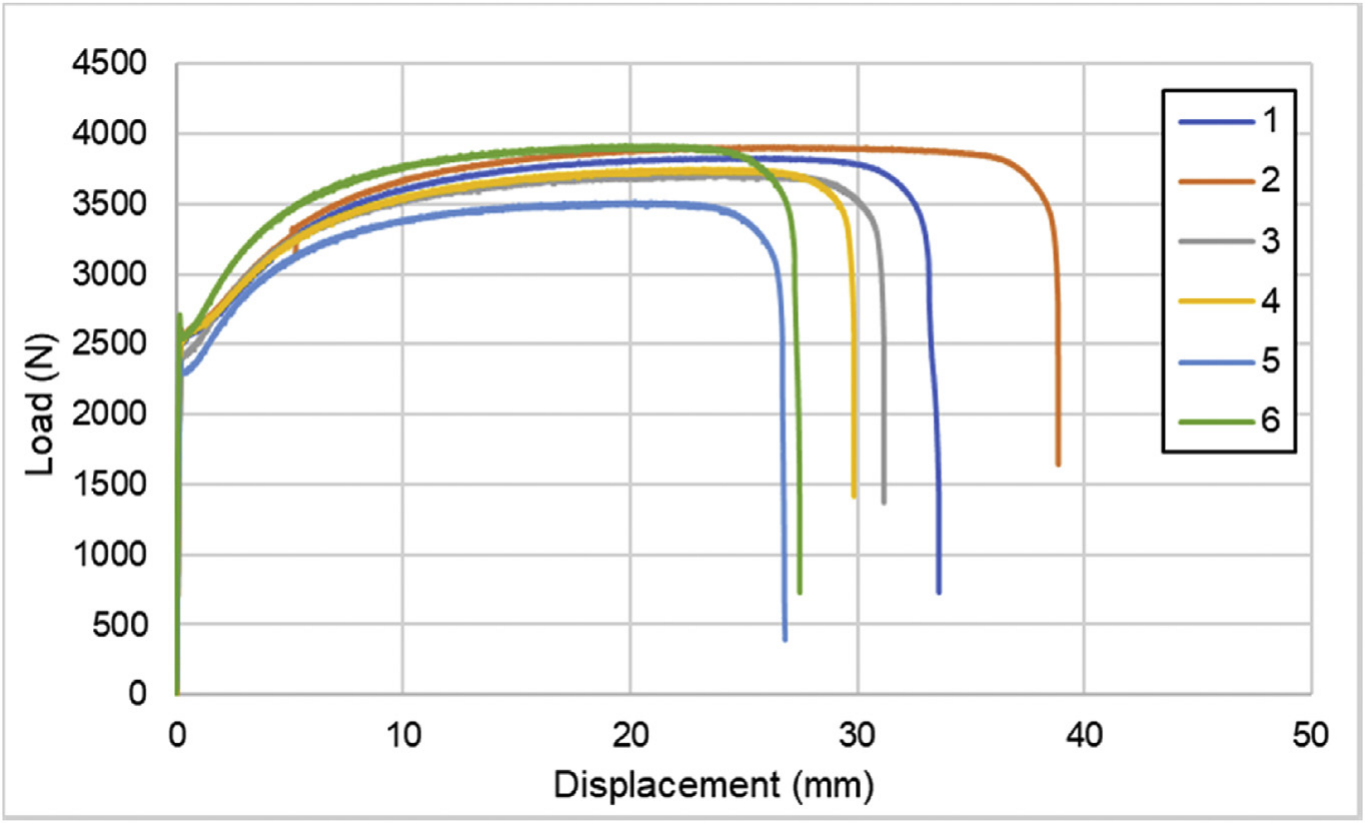
Load-displacement of SPCC plates.

**Fig. 2:**
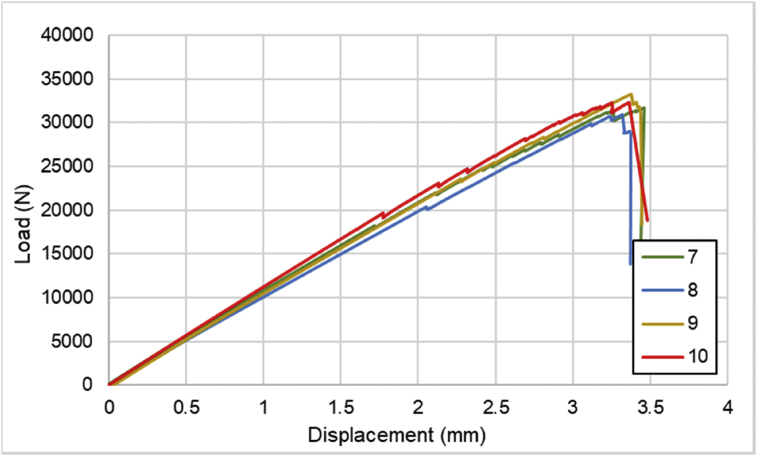
Load-displacement of [0]_4_ CFRP laminates.

**Fig. 3:**
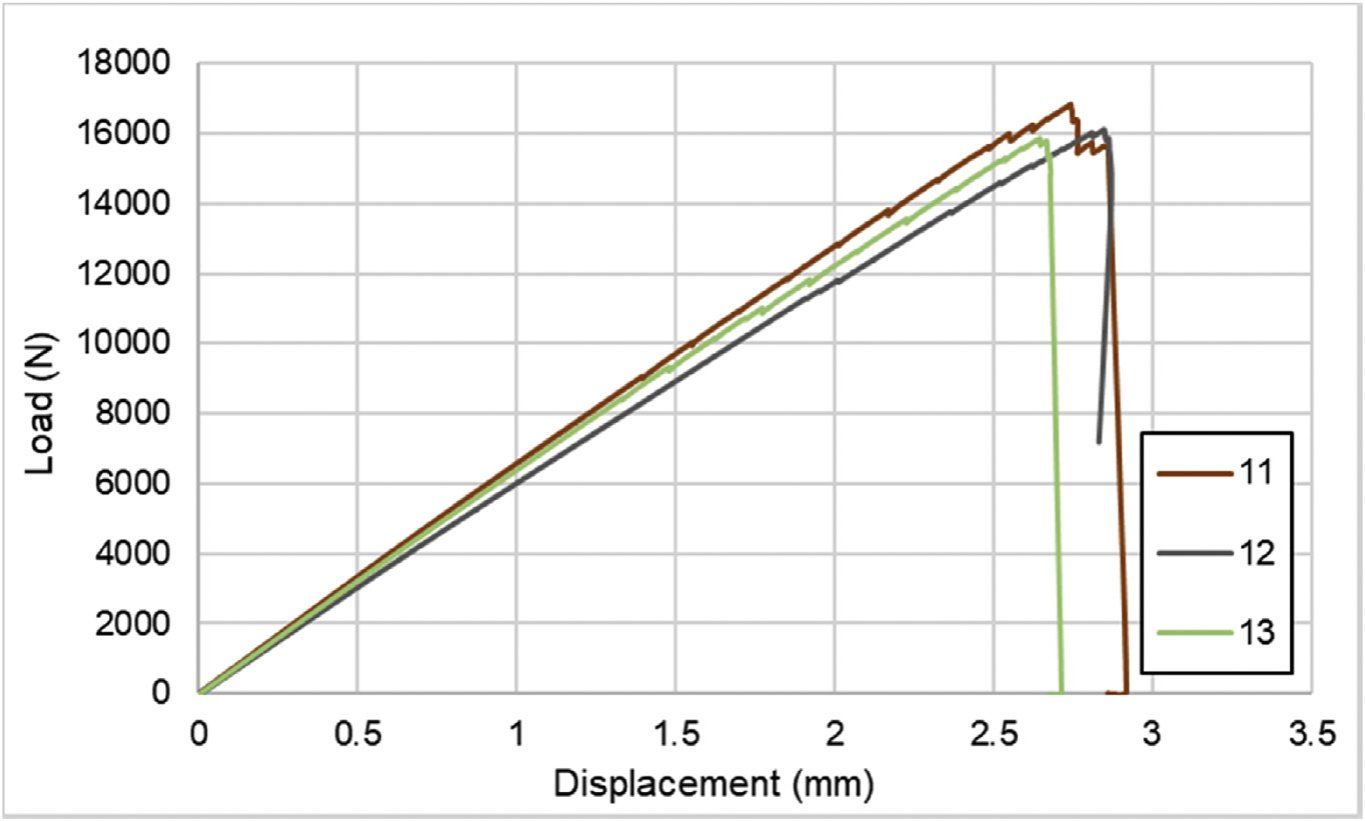
Load-displacement of [0]_2_ CFRP laminates.

**Fig. 4:**
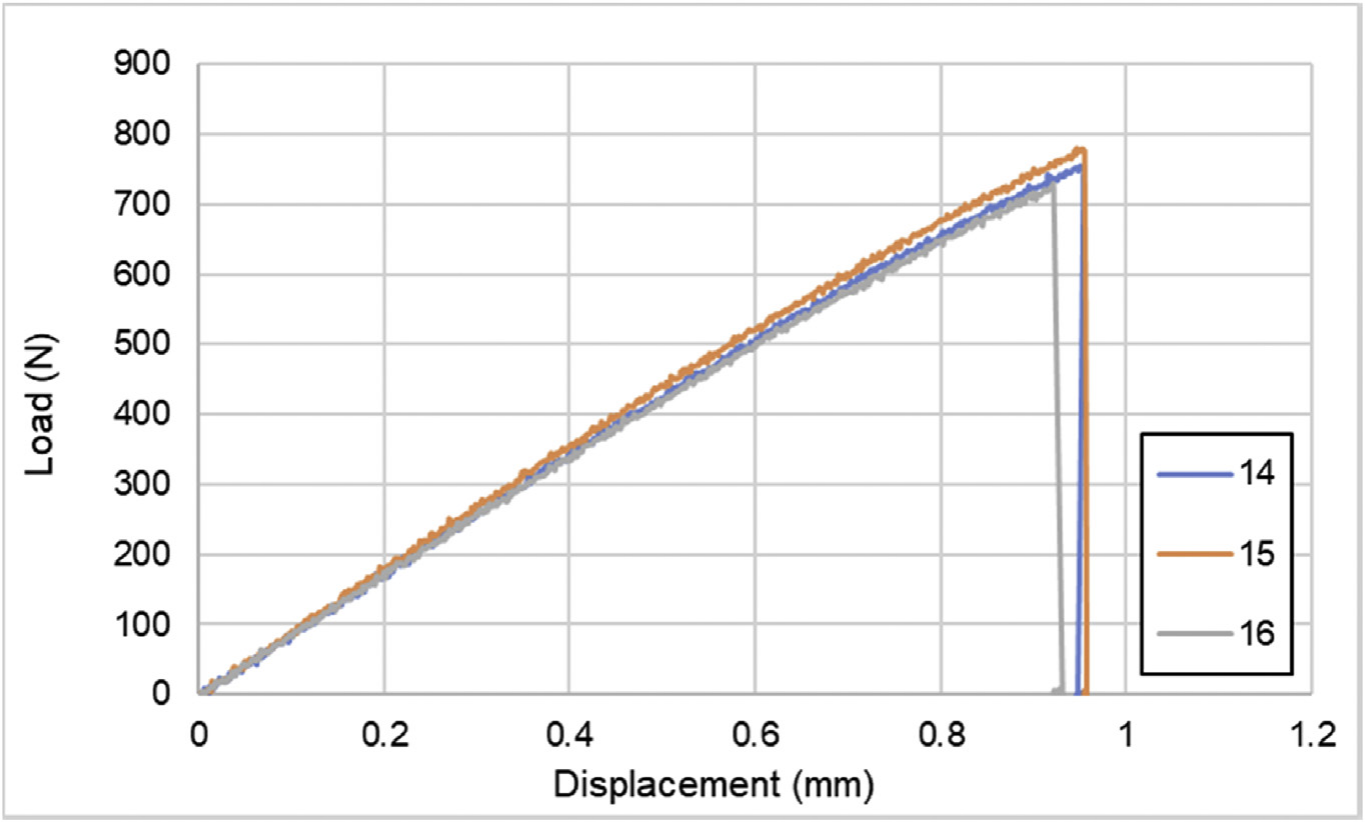
Load-displacement of [90]_4_ CFRP laminates.

**Fig. 5:**
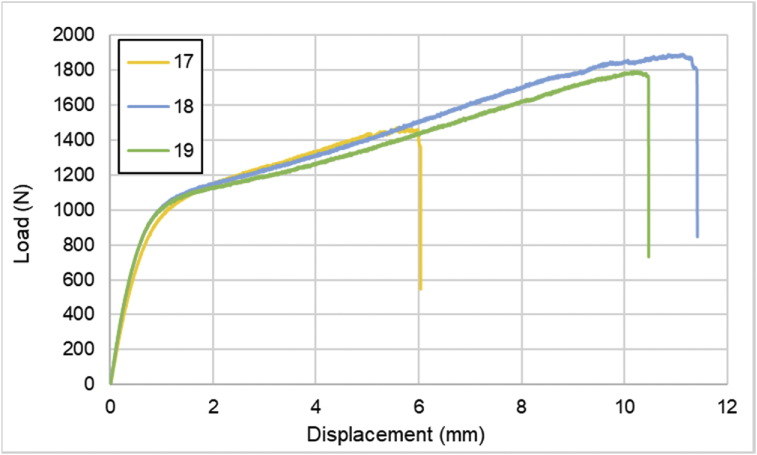
Load-displacement of [±45]_S_ CFRP laminates.

Comprehensive raw data of load-displacement values are available in the appendix. The data consist of specimens with several CFRPs and SPCCs can be seen in Table 1. Detailed dimension of all specimens can be seen in Table 2. Load-displacement of SPCC plate is shown in Fig. 1. Load-displacement of CFRP laminates with sequences of [0]_4_ can be seen in Fig. 2. Furthermore, for [0]_2_ CFRP laminate, load-displacement curves are illustrated in Fig. 3. The load-displacement performance of [90]_4_ and [±45]_S_ CFRP laminates are displayed in Figs. 4 and 5, respectively.

**Table 1:** List of specimens.

No.	Layups	Number of layers	
		CFRP	SPCC

1	SPCC	0	1
2	[0]_4_	4	0
3	[0]_2_	2	0
4	[90]_4_	4	0
5	[±45]_S_	4	0
6	[SPCC/0]_S_	2	2
7	[SPCC/0/0]_S_	4	2
8	[0/0/SPCC/0/0]	4	1
9	[±45/0]_S_	6	0
10	[0/0/90/90]_S_	8	0
11	[SPCC/±45/0]_S_	6	2
12	[SPCC/0/±45]_S_	6	2

**Table 2:** Dimension of all specimens.

No.	Specimen	Width (mm)	Mean Width (mm)	Thickness (mm)	Mean Thickness (mm)
1	SPCC-01	13.95	13.9667	0.804	0.8013
		13.95		0.802	
		14		0.798	
2	SPCC-02	14.15	14.2000	0.8	0.7990
		14.25		0.798	
		14.2		0.799	
3	SPCC-03	14.1	14.2167	0.802	0.8010
		14.25		0.801	
		14.3		0.8	
4	SPCC-04	14.2	14.0333	0.805	0.8043
		14		0.804	
		13.9		0.804	
5	SPCC-05	13.5	13.5333	0.802	0.8017
		13.5		0.802	
		13.6		0.801	
6	SPCC-06	15.1	15.1000	0.801	0.8023
		15.1		0.805	
		15.1		0.801	
7	[0]_4_-01	14.3	14.4500	0.667	0.6723
		14.35		0.679	
		14.7		0.671	
8	[0]_4_-02	13.85	13.9667	0.648	0.6467
		13.95		0.65	
		14.1		0.642	
9	[0]_4_-03	14.35	14.5000	0.664	0.6643
		14.5		0.673	
		14.65		0.656	
10	[0]_4_-04	14.4	14.3500	0.66	0.6697
		14.35		0.698	
		14.3		0.651	
11	[0]_2_-01	14.9	15.0000	0.32	0.3447
		15		0.378	
		15.1		0.336	
12	[0]_2_-02	13.4	13.4167	0.354	0.3563
		13.55		0.345	
		13.3		0.37	
13	[0]_2_-03	14.55	14.8667	0.34	0.3537
		14.9		0.363	
		15.15		0.358	
14	[90]_4_-01	15.25	15.2500	0.653	0.6623
		15.4		0.676	
		15.1		0.658	
15	[90]_4_-02	15.55	15.6667	0.657	0.6583
		15.7		0.662	
		15.75		0.656	
16	[90]_4_-03	15.05	15.1167	0.669	0.6670
		15.1		0.663	
		15.2		0.669	
17	[±45]_S_-01	14.1	14.1833	0.695	0.6850
		14.2		0.687	
		14.25		0.673	
18	[±45]_S_-02	15.5	15.4833	0.645	0.6320
		15.5		0.642	
		15.45		0.609	
19	[±45]_S_-03	15.65	15.9333	0.615	0.6230
		16.6		0.625	
		15.55		0.629	
20	[SPCC/0]_S_-01	15.2	15.0000	1.893	1.8933
		15		1.9	
		14.8		1.887	
21	[SPCC/0]_S_-02	14.8	14.5500	1.92	1.9010
		14.6		1.899	
		14.25		1.884	
22	[SPCC/0]_S_-03	14.55	14.6333	1.902	1.8850
		14.65		1.881	
		14.7		1.872	
23	[SPCC/0/0]_S_-01	15	14.8667	2.208	2.1810
		14.9		2.168	
		14.7		2.167	
24	[SPCC/0/0]_S_-02	15.05	14.8167	2.169	2.1823
		14.85		2.176	
		14.55		2.202	
25	[SPCC/0/0]_S_-03	15.5	15.2833	2.19	2.1973
		15.35		2.197	
		15		2.205	
26	[0/0/SPCC/0/0]-01	14.65	14.7500	1.442	1.4420
		14.75		1.444	
		14.85		1.44	
27	[0/0/SPCC/0/0]-02	14.8	14.9667	1.444	1.4473
		14.95		1.457	
		15.15		1.441	
28	[0/0/SPCC/0/0]-03	14.2	14.2500	1.443	1.4573
		14.2		1.467	
		14.35		1.462	
29	[±45/0]_S_-01	14	13.9167	0.957	0.9553
		13.9		0.967	
		13.85		0.942	
30	[±45/0]_S_-02	14.2	14.1833	0.943	0.9540
		14.1		0.951	
		14.25		0.968	
31	[±45/0]_S_-03	14	14.0000	0.957	0.9697
		14		0.969	
		14		0.983	
32	[±45/0]_S_-04	12.35	12.2833	0.975	0.9810
		12.3		0.99	
		12.2		0.978	
33	[0/0/90/90]_S_-01	14.2	14.2500	1.302	1.2990
		14.25		1.303	
		14.3		1.292	
34	[0/0/90/90]_S_-02	14.3	14.3167	1.308	1.3127
		14.35		1.308	
		14.3		1.322	
35	[0/0/90/90]_S_-03	14.35	14.3500	1.306	1.3097
		14.35		1.315	
		14.35		1.308	
36	[SPCC/±45/0]_S_-01	15.55	15.4500	2.59	2.5750
		15.4		2.567	
		15.4		2.568	
37	[SPCC/±45/0]_S_-02	13.65	13.5667	2.566	2.5813
		13.55		2.597	
		13.5		2.581	
38	[SPCC/±45/0]_S_-03	14.5	14.5000	2.557	2.5647
		14.5		2.598	
		14.5		2.539	
39	[SPCC/±45/0]_S_-04	14.05	14.5667	2.51	2.5033
		15		2.501	
		14.65		2.499	
40	[SPCC/0/±45]_S_-01	14.7	14.6667	2.583	2.5937
		14.55		2.6	
		14.75		2.598	
41	[SPCC/0/±45]_S_-02	12.75	12.7833	2.589	2.5940
		12.85		2.593	
		12.75		2.6	
42	[SPCC/0/±45]_S_-03	12.85	12.8667	2.617	2.5940
		12.85		2.55	
		12.9		2.615

**Fig. 6:**
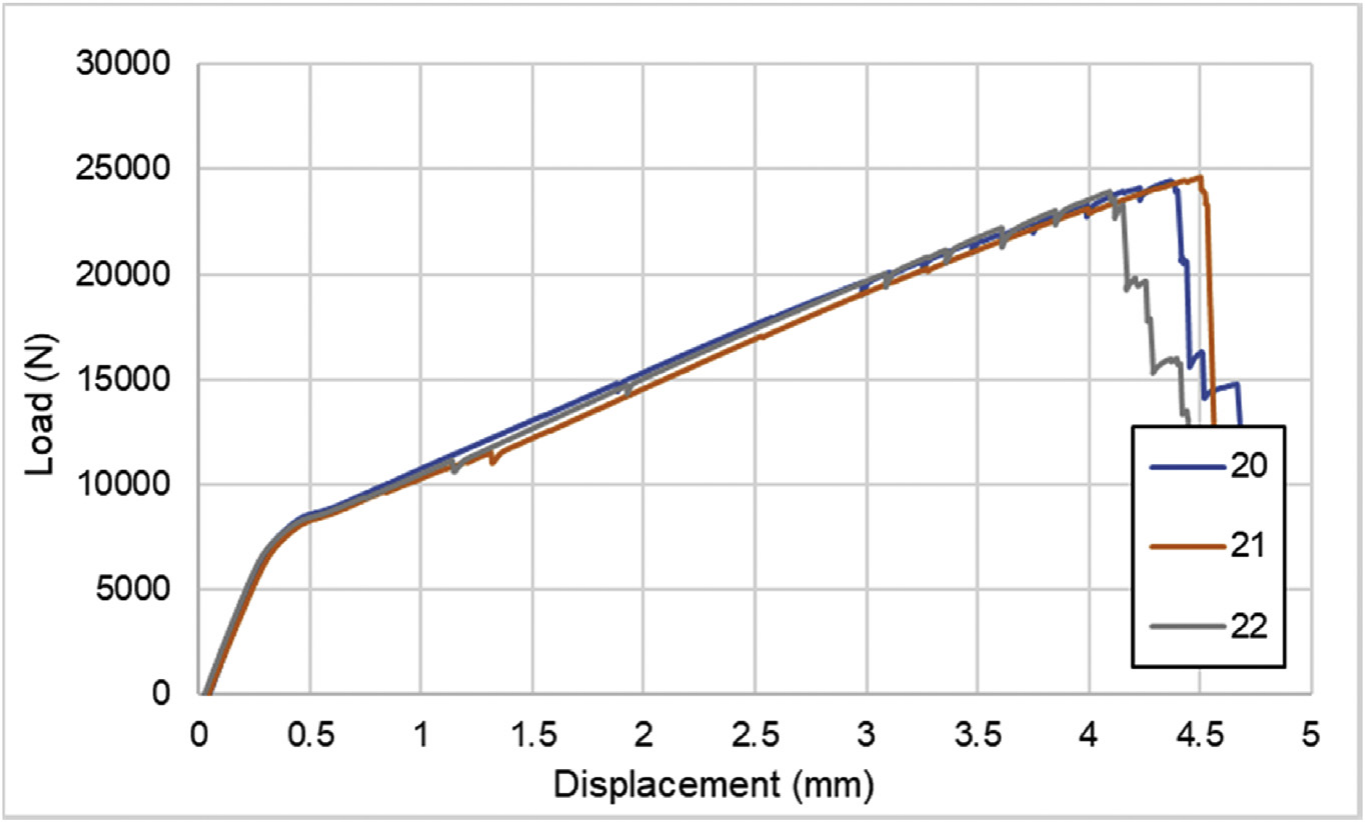
Load-displacement of [SPCC/0]_S_ CFRP-SPCC hybrid laminates.

**Fig. 7:**
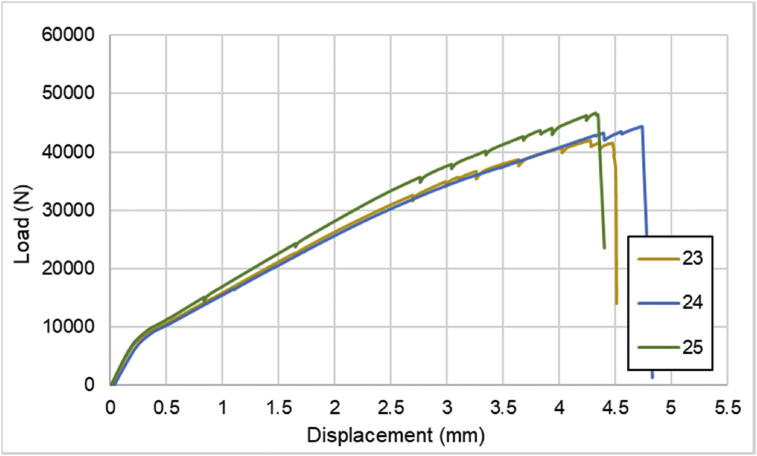
Load-displacement of [SPCC/0/0]_S_ CFRP-SPCC hybrid laminates.

**Fig. 8:**
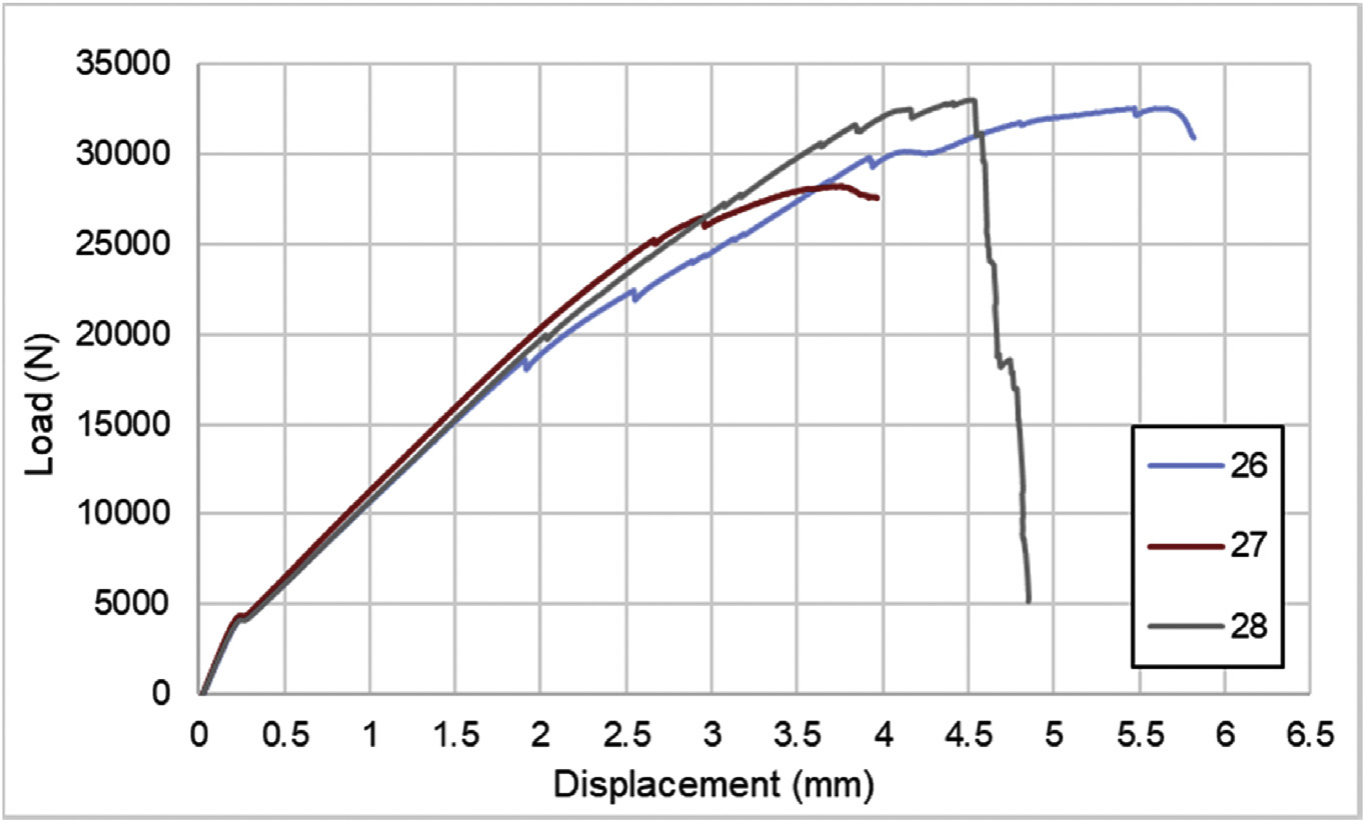
Load-displacement of [0/0/SPCC/0/0] CFRP-SPCC hybrid laminates.

**Fig. 9:**
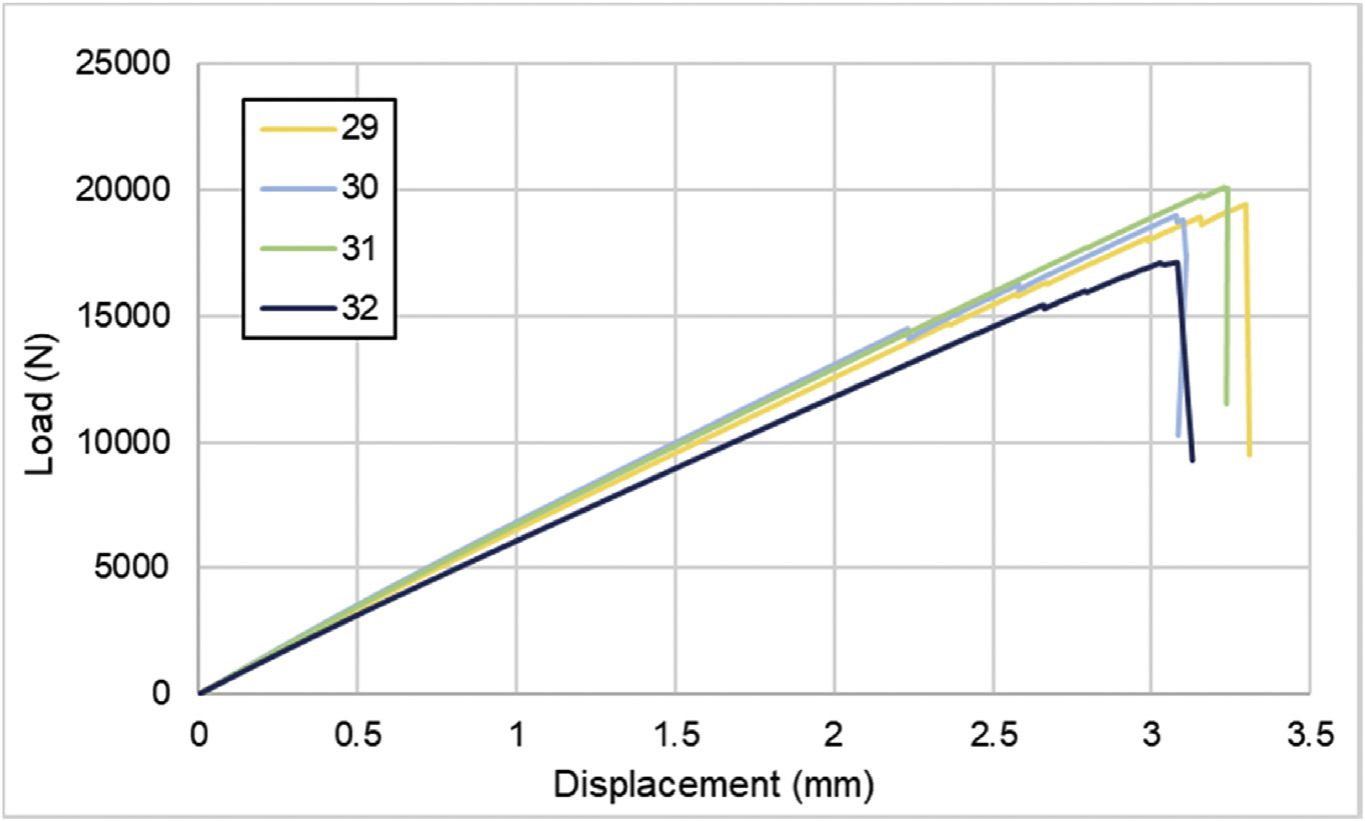
Load-displacement of [±45/0]_S_ CFRP laminates.

**Fig. 10:**
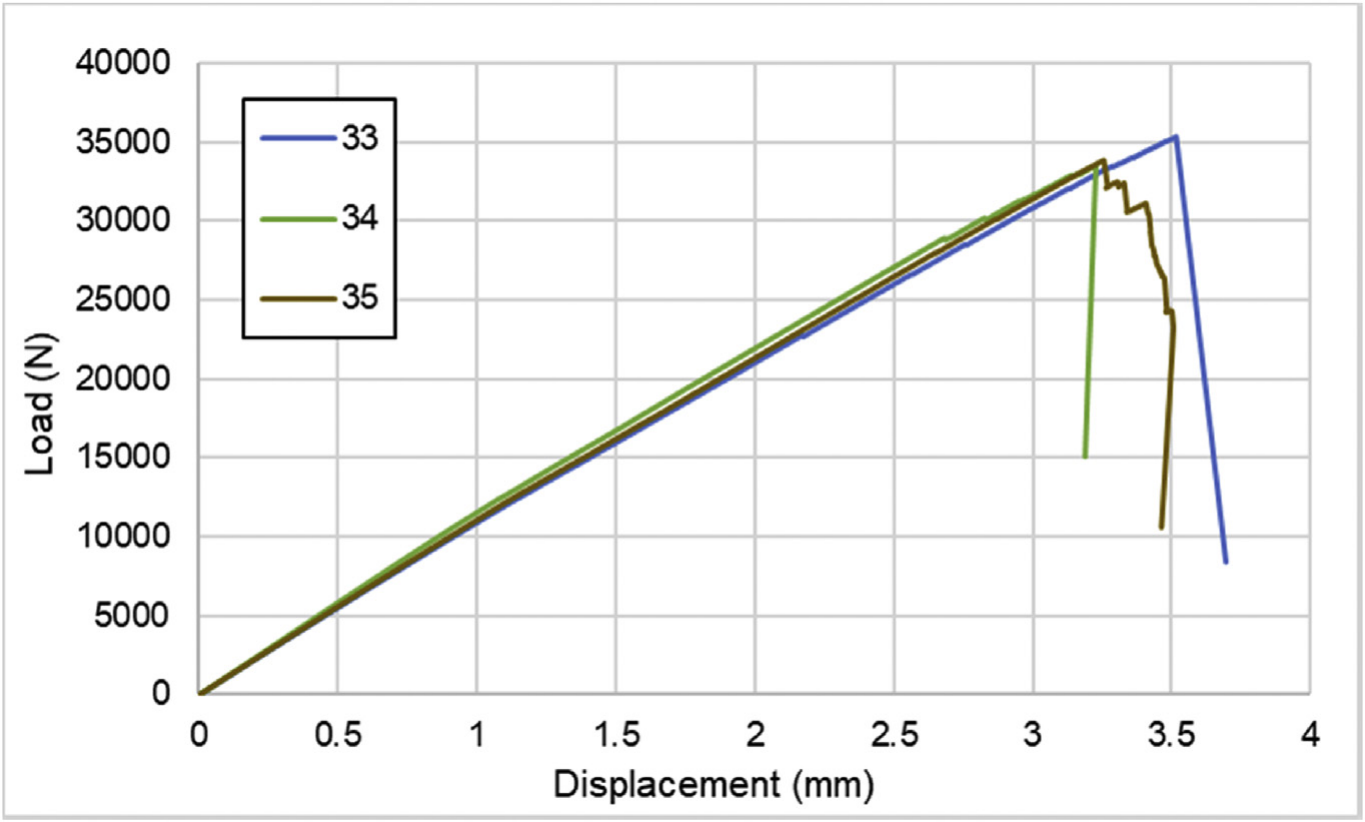
Load-displacement of [0/0/90/90]_S_ CFRP laminates.

**Fig. 11:**
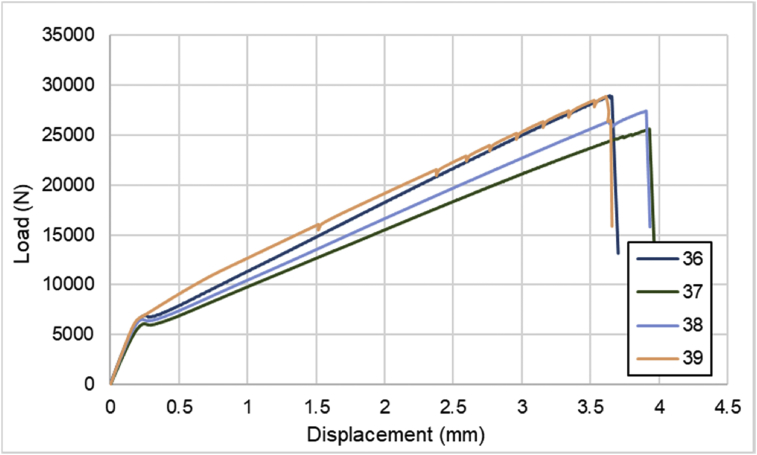
Load-displacement of [SPCC/±45/0]_S_ CFRP-SPCC hybrid laminates.

**Fig. 12:**
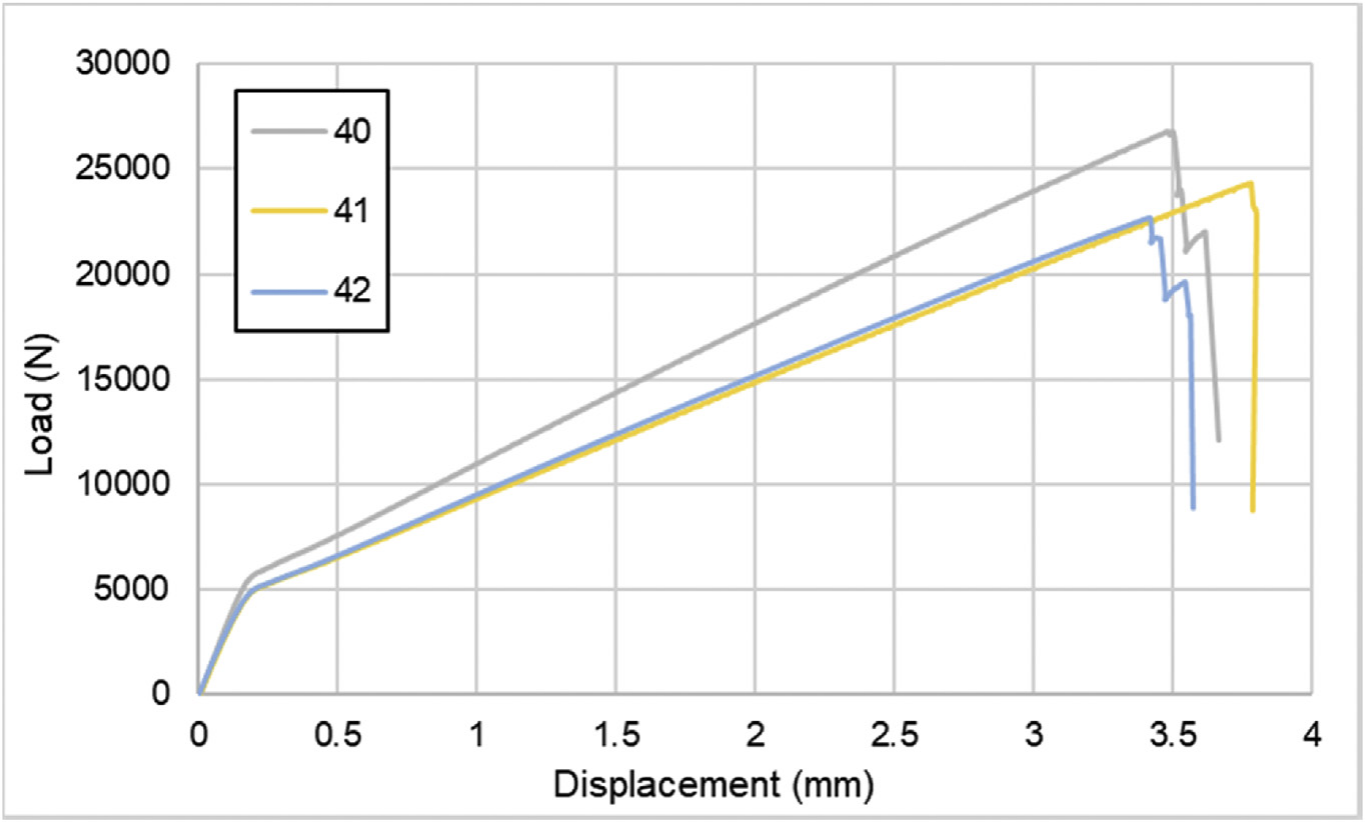
Load-displacement of [SPCC/0/±45]_S_ CFRP-SPCC hybrid laminates.

For hybrid laminates that consist of SPCC and 0°-layer of CFRP laminate are presented in Fig. 6, Fig. 7, Fig. 8 with the sequences of [SPCC/0]_S_, [SPCC/0/0]_S_, and [0/0/SPCC/0/0]. Moreover, for combination of 0°-layer and non 0°-layer of CFRP, Fig. 9 with 4 specimens, shows load-displacement curves of [±45/0]_S_. Fig. 10 shows the load-displacement performance of [0/0/90/90]_S_. For the last two different combinations, load-displacement curves can be seen in Figs. 11 and 12 with [SPCC/±45/0]_S_, and [SPCC/0/±45]_S_ hybrid CFRP-SPCC laminates.

## 2 Experimental design, materials, and methods

### 2.1 Specimen preparation and test

**Fig. 13:**
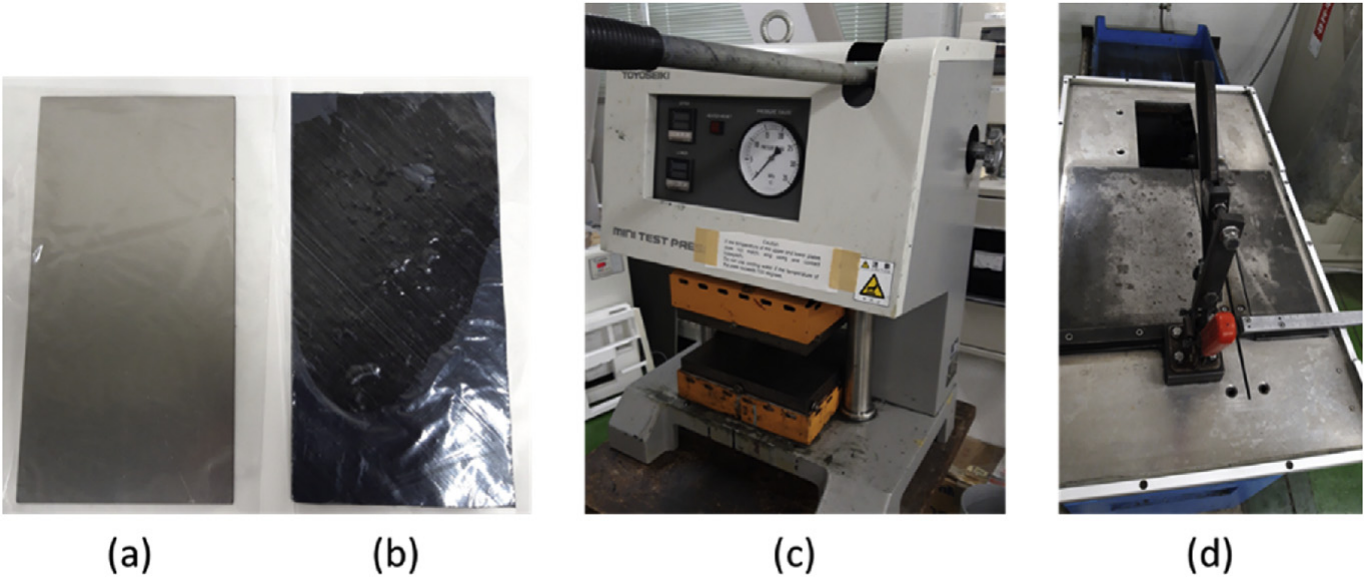
(a) SPCC plate, (b) Prepreg CFRP, (c) Hot press machine, and (d) Cutting machine.

The steel used in the research is called Steel Plate Cold Commercial (SPCC), or equivalent to JIS G 3141 with 0.8 mm of thickness. SPCC commonly used in structures applications and automobile parts [1]. Prepreg CFRP T800 from Toray Industries Inc. were manufactured alongside with SPCC directly by using hand lay-up technique. Curing process were used hot press machine with 130 °C for 3 h in room temperature condition (25 °C) to ensure all resin completely cured. The specimen then cut based on ASTM D3039 by using cutting machine. Fig. 13 show materials used in the study, hot press machine for curing process, and cutting machine to cut the specimens.

The steel used in this research is called Steel Plate Cold Commercial (SPCC), or equivalent to JIS G 3141 with 0.8 mm of thickness. SPCC is commonly used in structures applications and automobile parts [1]. Prepreg CFRP T800 from Toray Industries, Inc. were manufactured alongside with SPCC directly by using hand lay-up technique. Curing process was done by using hot-press machine with 130 °C for 3 h to ensure all resin completely cured. After curing, cutting process, sample preparations and testing were done in the room temperature (25 °C). The specimens were then cut based on ASTM D3039 by using cutting machine. Fig. 13 show materials used in the study, hot press machine for curing process, and cutting machine to cut the specimens.

**Fig. 14:**
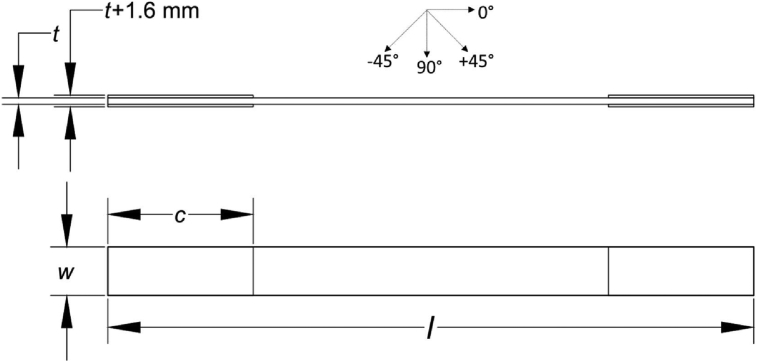
Specimen dimension [2].

**Fig. 15:**
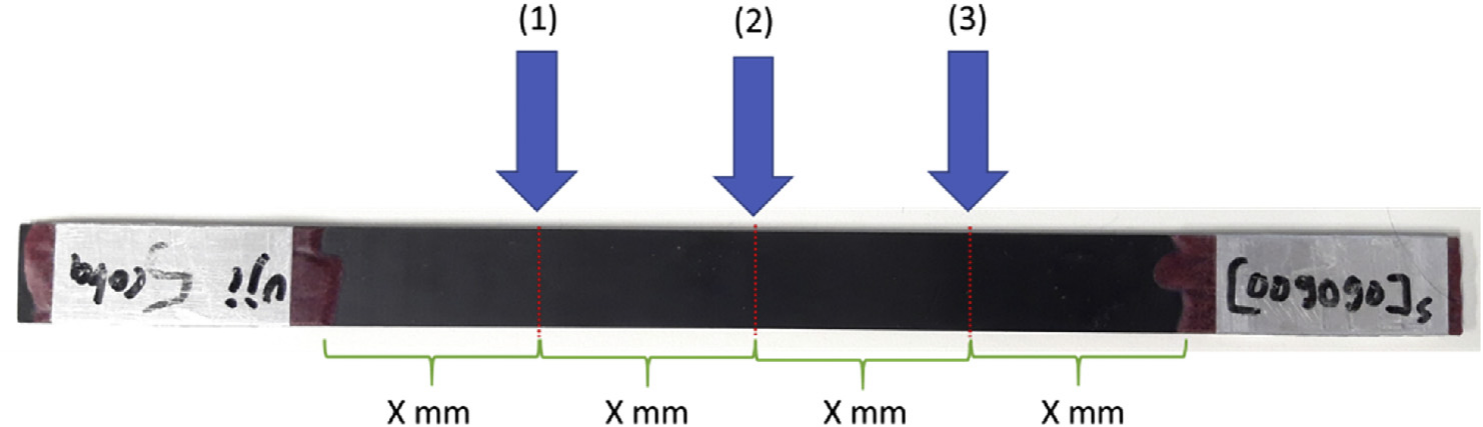
Specimen spots for thickness and width measurement.

**Fig. 16:**
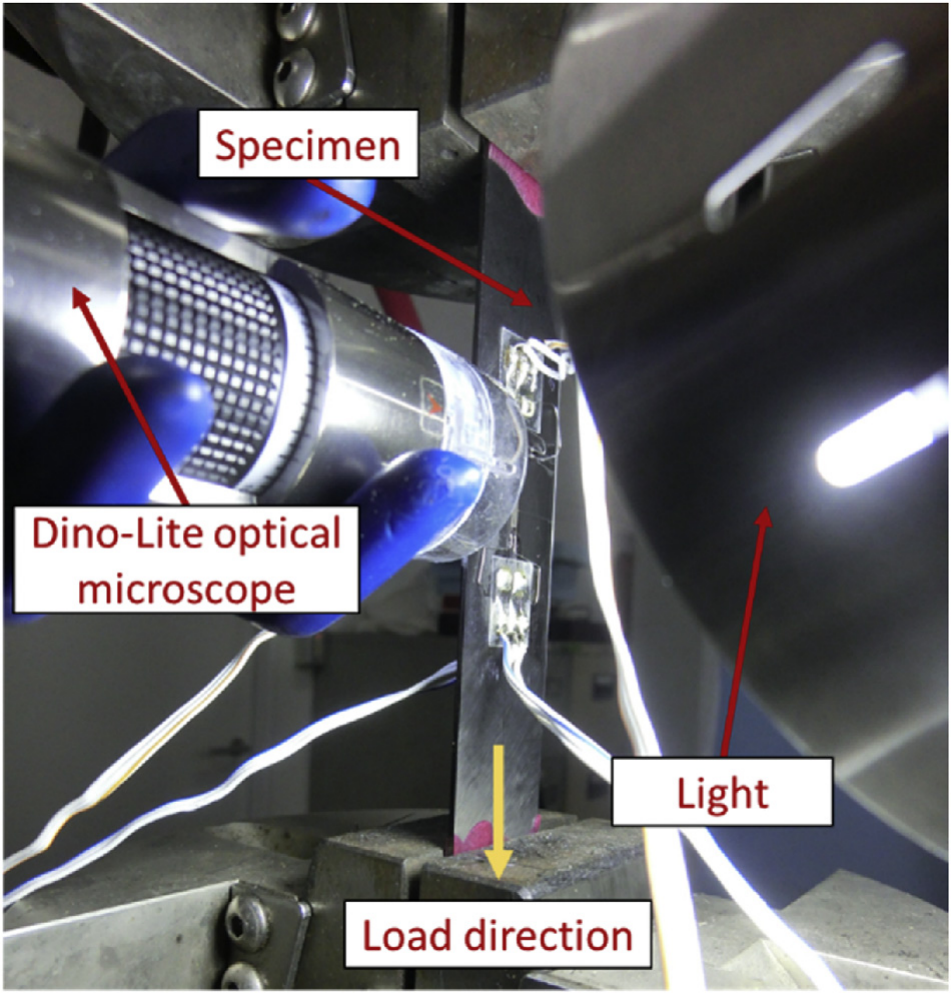
Experimental setup.

Before testing specimens, they were attached to 0.5 mm of aluminium tab with 40–50 mm length at both ends. The detailed specimen's dimension can be seen in Fig. 14 where *t* is the specimen thickness (mm), *w* is specimen width (mm), *c* is tab length (45 mm), *l* is total specimen length (200 mm). Data of specimen thickness and width are shown in Table 2. At least 3 different positions were required to measure specimen thickness and width. The detailed measurement method is illustrated in Fig. 15. Tensile test was conducted by using an Instron servo-hydraulic Universal Testing Machine (UTM) 8802. During tensile loading, load-displacement were recorded automatically until the failure of specimens. To investigate the condition of side surface of laminates during tensile loading, a Dino-Lite optical microscope was used. Detailed experimental setup is shown in Fig. 16.

### 2.2 Note from the experiment

• To increase the bonding strength between CFRP and SPCC, sandpaper P120 can be used to increase SPCC surface roughness.
• After sandpaper applied, ethanol was used with a clean tissue to remove all debris and SPCC tiny residual object from the SPCC surface. Make sure to clean all the surface and remove all the pollutants.
• To avoid pollutant attached on the material surface and hands, lab gloves can be used.
• Placed specimen in the hot press machine before the machine is started.
• Use heat resistance gloves to remove the specimen from hot press machine.
• Do not directly cut the sample while the sample is not properly cool and still in cooling process. At least wait 4 h to make sure the sample is properly cured and cool.
• Carefully to use cutting machine. Make sure to use gloves and lab glasses to protect the eyes.
• Keep distance during tensile loading is in progress since the delamination of CFRP may cause injury since it usually forms as sharp debris.
